# Supporting healthy lifestyles for First Nations women and communities through co-design: lessons and early findings from remote Northern Australia

**DOI:** 10.3389/fcdhc.2024.1356060

**Published:** 2024-05-28

**Authors:** Tara Dias, Diana MacKay, Karla Canuto, Jacqueline A. Boyle, Heather D’Antoine, Denella Hampton, Kim Martin, Jessica Phillips, Norlisha Bartlett, H. David Mcintyre, Sian Graham, Sumaria Corpus, Christine Connors, Leisa McCarthy, Renae Kirkham, Louise J. Maple-Brown

**Affiliations:** ^1^ Menzies School of Health Research, Charles Darwin University, Darwin, NT, Australia; ^2^ Department of Endocrinology, Royal Darwin Hospital, Darwin, NT, Australia; ^3^ College of Medicine and Public Health, Flinders University , Melbourne, VIC, Australia; ^4^ Eastern Health Clinical School, Monash University, Melbourne, VIC, Australia; ^5^ School of Public Health, University of Queensland, Brisbane, QLD, Australia; ^6^ Central Australian Aboriginal Congress, Alice Springs, NT, Australia; ^7^ Aboriginal and Torres Strait Islander Advisory Group, Diabetes Across the Lifecourse: Northern Australia Partnership, Darwin, NT, Australia; ^8^ Women’s Cultural Hub, Mala’la Community Wellness Centre, Mala’la Aboriginal Health Corporation, Maningrida, NT, Australia; ^9^ Mater Research, The University of Queensland, Brisbane, QLD, Australia; ^10^ Northern Territory Department of Health, Darwin, NT, Australia; ^11^ Anyinginyi Health Aboriginal Corporation, Tennant Creek, NT, Australia

**Keywords:** diabetes, gestational diabetes, diabetes in pregnancy, First Nations, lifestyle modifications, co-design, participatory research, experience-based co-design

## Abstract

**Background:**

The period before, during, and after pregnancy presents an opportunity to reduce diabetes-related risks, which in Australia disproportionately impact Aboriginal and Torres Strait Islander women. Collaboration with Aboriginal and Torres Strait Islander women/communities is essential to ensure acceptability and sustainability of lifestyle modifications. Using a novel co-design approach, we aimed to identify shared priorities and potential lifestyle strategies. We also reflected on learnings from this approach.

**Methods:**

We conducted 11 workshops and 8 interviews at two sites in Australia’s Northern Territory (Central Australia and Top End), using experience-based co-design (EBCD) and incorporating principles of First Nations participatory research. Workshops/interviews explored participant’ experiences and understanding of diabetes in pregnancy, contextual issues, and potential lifestyle strategies. Participants included three groups: 1) Aboriginal and Torres Strait Islander women of reproductive age (defined as aged 16-45 years); 2) Aboriginal and Torres Strait Islander community members; and 3) health/community services professionals. The study methodology sought to amplify the voices of Aboriginal women.

**Findings:**

Participants included 23 women between ages 16-45 years (9 with known lived experience of diabetes in pregnancy), 5 community members and 23 health professionals. Key findings related to identified priority issues, strategies to address priorities, and reflections on use of EBCD methodology. Priorities were largely consistent across study regions: access to healthy foods and physical activity; connection to traditional practices and culture; communication regarding diabetes and related risks; and the difficulty for women of prioritising their health among competing priorities. Strategies included implementation of a holistic women’s program in Central Australia, while Top End participants expressed the desire to improve nutrition, peer support and community awareness of diabetes. EBCD provided a useful structure to explore participants’ experiences and collectively determine priorities, while allowing for modifications to ensure co-design methods were contextually appropriate. Challenges included the resource-intensive nature of stakeholder engagement, and collaborating effectively with services and communities when researchers were “outsiders”.

**Conclusions:**

A hybrid methodology using EBCD and First Nations participatory research principles enabled collaboration between Aboriginal women, communities and health services to identify shared priorities and solutions to reduce diabetes-related health risks. Genuine co-design processes support self-determination and enhance acceptability and sustainability of health strategies.

## 1 Introduction

Diabetes in pregnancy, encompassing gestational diabetes (GDM) and pre-existing diabetes, increases long-term diabetes-related risks for both mothers and infants (1–4). In countries with a history of colonisation, the intergenerational impact of diabetes in pregnancy is a major contributor to the disproportionately high prevalence of type 2 diabetes (T2D) among First Nations peoples (5). In Australia’s Northern Territory (NT), the incidence of diabetes in pregnancy among Aboriginal and Torres Islander women has substantially increased over recent decades (6). There is an urgent need to reduce the risks associated with diabetes in pregnancy to arrest the growing epidemic of T2D among Aboriginal and Torres Strait Islander peoples (7). It has been recognised that strategies to reduce these risks should embrace the strengths of Aboriginal and Torres Strait Islander culture, including the benefits of connection to family, community and country (8–10).

The pervasive impacts of colonisation, however, create multiple barriers to reducing the risks associated with diabetes in pregnancy (5). Colonisation has particularly influenced the social determinants of health, resulting in poverty, housing and food insecurity, and disruptions to education and employment (11, 12). Health services are often provided by non-Indigenous clinicians in a Western model, with challenges in cross-cultural communication leading to messages relating to diabetes in pregnancy being understood only at a superficial level (8). In this setting, there is limited engagement with postpartum follow-up after a pregnancy complicated by diabetes; only 52% of Aboriginal women in the NT with GDM in 2013-14 had recommended glucose testing in the 12 months postpartum (13). The needs of Aboriginal and Torres Strait Islander women with diabetes in pregnancy have not consistently or comprehensively been met in the Western-dominated Australian context, creating an impetus to use other approaches to develop and implement strategies to reduce diabetes-related risks.

The right of First Nations peoples to exercise self-determination, including choices regarding health, is enshrined in international law (14). Similarly, health consumer participation (at the individual, service, and policy level) is increasingly considered a right and an expectation (15). Participatory research approaches have the potential to strengthen translational research through ensuring lived experience is valued (16). In the First Nations context, use of these approaches can result in the creation of respectful spaces for traditional knowledges and community-based decision making (17). ‘Co-design’ has become a shorthand term for a range of participatory approaches aiming to address inequities and transform hierarchical power dynamics traditionally associated with research (18). These approaches align with First Nations research methodologies, which acknowledge the communal creation and analyses of knowledges (19), and which have operated traditionally outside spheres of academic recognition (20). Experience-based co-design is increasingly used in Aboriginal and Torres Strait Islander health research however there is limited evidence regarding this methodology in our context.

The DIABETES Across the LIFECOURSE: Northern Australia Partnership (‘the Partnership’) first commenced in the Northern Territory in 2011 with a focus on diabetes in pregnancy. Since 2020, the Partnership has been collaborating with Aboriginal and Torres Strait Islander women, families, communities and health services in a multi-phase study to implement and evaluate co-designed strategies to address the risks before, during and after a pregnancy complicated by diabetes. In the phase of the study reported here, we aimed to identify priorities and potential strategies at study sites in Australia’s Northern Territory. We also reflect on our learnings relating to the process of co-design.

## 2 Methods and research paradigm

### 2.1 Methodology and incorporation of First Nations research principles

**Figure 1:**
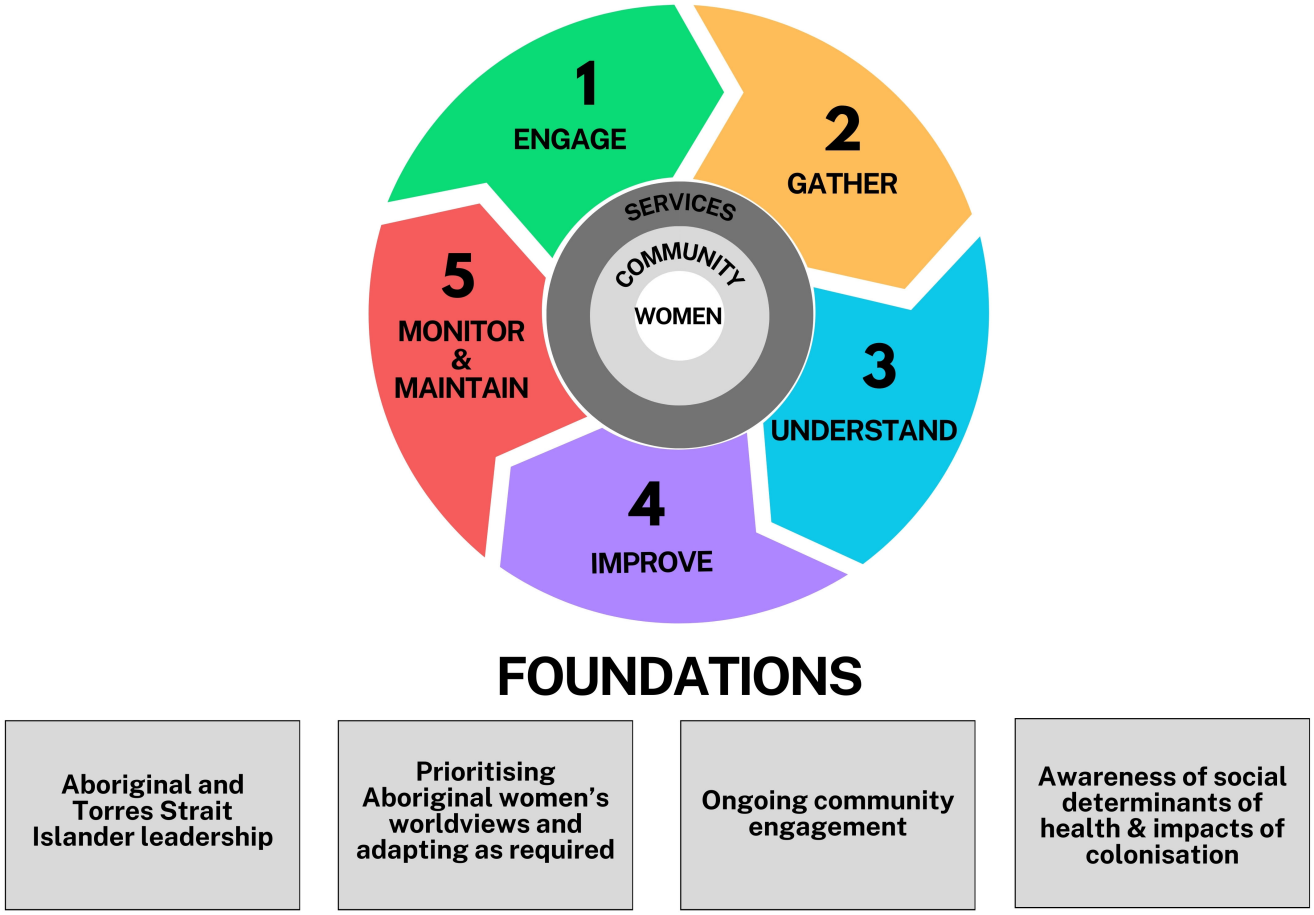
Hybrid methodology incorporating experience-based co-design and First Nations participatory research principles (Adapted with permission from Dadwa & Knight, 2017).

The study uses a transformative paradigm. Key defining characteristics include an understanding that power and privilege play a role in determining which knowledge is ‘valid’ and that dialogue with participants is necessary to fully understand the realities of participants (21). A transformative paradigm considers issues of power and social justice, requiring ongoing self-reflection by researchers to build interactive and empowering relationships with participants (21). This study’s methods are a hybrid of experience-based co-design (EBCD) with consideration of principles of First Nations participatory approaches. EBCD, derived from the design sciences, is a service-user driven design process focussing on improving (consumer) experiences (22) and is increasingly used in the health sector (23). EBCD is characterised by distinct stages; engagement activities, interviews, and workshops are used during this process (see **Figure 1**). Implementation approaches of EBCD projects vary widely but common principles include participation (inviting participants into a collaborative process); development (an iterative, evolving process); transformation of power relationships which enable ownership; and having practical, tangible outcomes that benefit participants (23).

First Nations academics have described traditions that honour iterative research approaches, requiring a foundation of respectful relationships, and acknowledgement that knowledge is communally created and owned (19). Participatory processes have traditionally guided decision making in First Nations communities (20). To support enhanced cultural responsiveness of our approach, we augmented the structures of EBCD with the following principles: inclusion of First Nations community members/stakeholders and leadership throughout the process at study sites; prioritising the voices of First Nations women; and supporting culturally responsive two-way learning and First Nations leadership (with First Nations investigators, staff, and governance structures).

### 2.2 Study governance and the Aboriginal and Torres Strait Islander Advisory Group

Study staff and investigators were guided by input from the Partnership’s Aboriginal and Torres Strait Islander Advisory Group, who provided advice on a quarterly basis regarding engagement and cultural considerations. This group guides the governance of the Partnership and set the research priorities, contribute to design of projects and approve or decline research proposals before grant or ethics submissions. Co-authors SG, DH and SC contributed as members of the Advisory Group. As outlined under “Methodology and Principles”, First Nations leadership and voices were prioritised throughout the study. The study was also guided by the Partnership’s Steering Committee, comprising investigators and key stakeholders, who advised on study design and implementation, and advised on the effectiveness and feasibility of strategies proposed through the co-design process. Aboriginal and Torres Strait Islander investigators, in addition to contributing a cultural lens, provided expertise in midwifery, health promotion, and food security.

### 2.3 Setting

This study was situated in two sites in Australia’s Northern Territory (NT), one in each of the NT’s regions, being Top End and Central Australia. Residents identifying as Aboriginal and Torres Strait Islander people comprise 27% of the NT’s population, compared to 3.2% nationally (24, 25). Over 200 languages, including Aboriginal and Torres Strait Islander languages and those of migrant populations, are spoken across the NT (24). In 2020, approximately 28% of the 1200 births to Aboriginal and Torres Strait Islander women annually were complicated by diabetes (26). At commencement of this study, the Partnership had established relationships with both study sites, including through collaboration on prior qualitative research which informed this study (8). Governance approvals were sought and received from the boards of the Aboriginal Community-Controlled Health Service in each community (Central Australian Aboriginal Congress and Mala’la Aboriginal Health Corporation in the Top End). Where appropriate, research approvals were sought and received from Land Councils.

In Central Australia, the study was conducted in the region’s major centre of Alice Springs, which has a population of 25,000. Residents can access primary healthcare through the local Aboriginal Community Controlled Health Service (Central Australian Aboriginal Congress) or through private providers and attend the Alice Springs Hospital for specialist care.

The study site in the Top End was a remote community in Arnhem land with a population of approximately 2500 people. Healthcare is provided locally by an Aboriginal Community Controlled Health Service, Mala’la Health Service, with specialist input from the region’s referral hospital. The community is separated from the referral hospital by over 500 km, or a 9-hour road journey, which is frequently impassable during the monsoonal wet season.

### 2.4 Research team and positionality

The co-design process for this study was co-led by DM and TD. DM is a non-Indigenous, first-generation Australian woman with expertise in mixed-methods, including qualitative research with Aboriginal women, and has worked as an endocrinologist in the NT for five years. TD is a non-Indigenous woman originally from the United States, who has worked in systemic advocacy, service design, and policy in Fiji and the NT for the past decade. Participant engagement, workshop facilitation, and data collection were undertaken by regional teams (KM and DH in Central Australia; JP, TD and NB in Top End). KM is a non-Indigenous woman with a background in physiotherapy, who has three years of experience in research using collaborative methods relating to diabetes with Aboriginal people in Central Australia. DH is an Aboriginal woman (Warumunga and Nukuna/Gothaka) and has been working as a midwife in Central Australia for 11 years. JP is a Burarra woman, raised and based in Top End, with experience in community development and women’s services. NB is a Bardi and Jabirr-Jabirr woman from the Kimberley and has worked in the remote context for the past nine years, and with the Diabetes Partnership (in Darwin) for the past four, focused on stakeholder engagement. In addition to employing Aboriginal women as team members (NB, DH, JP), regional teams worked with female Aboriginal community/clinical leaders throughout the project to support participant engagement and cultural responsiveness. Research team members participated in facilitation training prior the commencement of data collection activities.

### 2.5 Participant recruitment

Research activities reported on in this paper occurred in Central Australia April 2022-October 2023 and in Top End February 2023-October 2023. Three groups of participants were invited to participate: 1) Aboriginal and Torres Strait Islander women of reproductive age (defined as aged 16 to 45 years) 2) Aboriginal and Torres Strait Islander community members including Elders, carers, and other community members and 3) community service professionals and health professionals involved in the care of women with diabetes during pregnancy. Participant numbers are outlined in **Table 1**. Participants were excluded if they were unable to provide informed consent or were under 16 years of age.

**Table 1:** Co-design participants.

		Women (with DIP)	Women (without/not known to have DIP)	Community/Elder	Health/Community services professional
Central Australia participants	Workshops	6	11	0	18
	Interviews	0	0	0	0
	Total participants Central Australia	6	11	0	18
Top End participants	Workshops only	2	3	4	1
	Interviews only	1	0	0	2
	Workshop and interview	0	0	1**	2**
	Total participants Top End	3	3	5	5
Total participants		9	14*	5	23
		23		5	23

DIP, diabetes in pregnancy.

All women, community members and Elders identified as Aboriginal and/or Torres Strait Islander. Many participants participated in more than one data collection activity due to the iterative nature of the project.

*5 of the women without a personal experience of diabetes in pregnancy have a close family member living with diabetes.

**These participants opted to participate in both interviews and workshops due to preference and/or availability.

**Figure 2:**
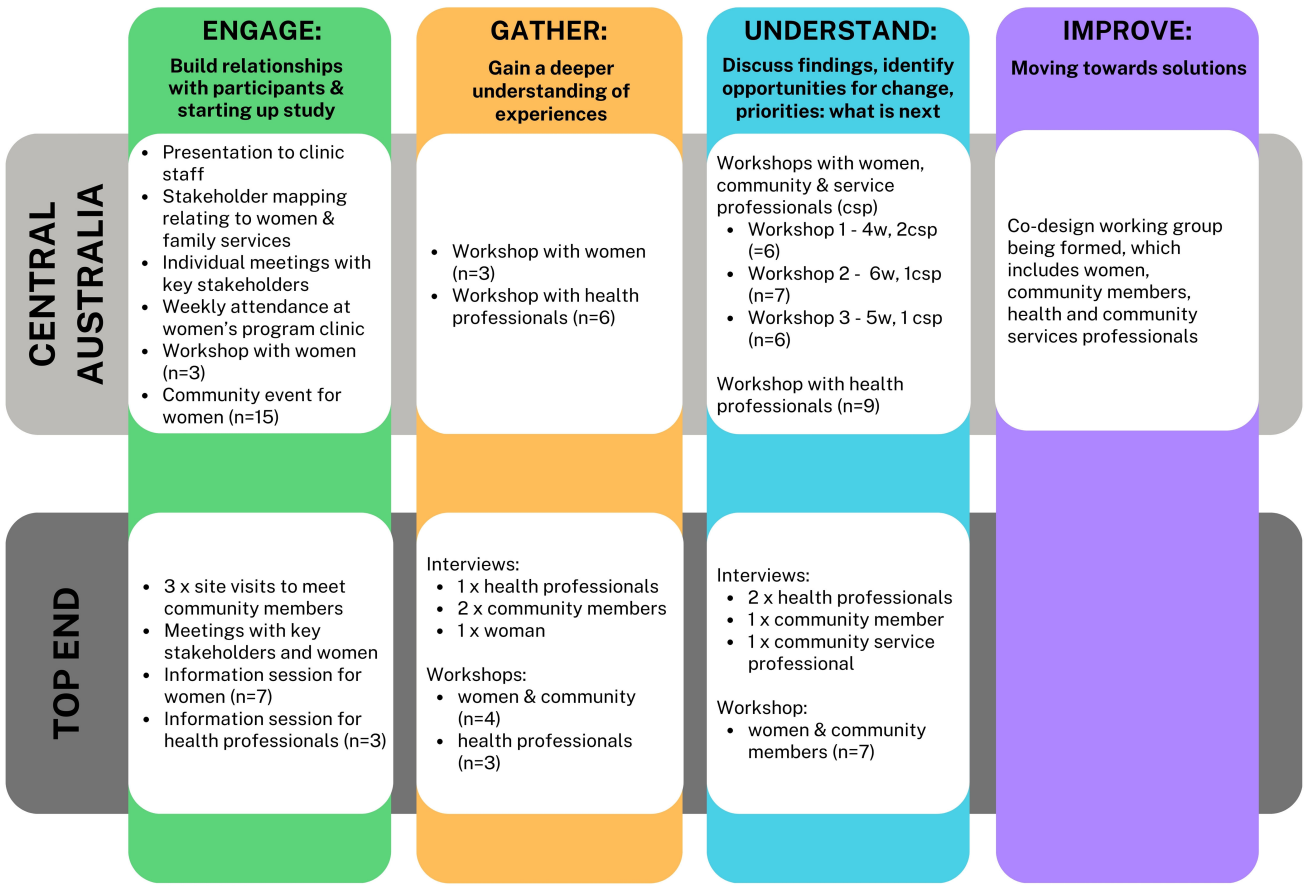
Data collection activities and participant numbers according to site and by EBCD stage.

**Figure 2** and the **Supplementary Table** provides more detail about the format and timelines associated with data collection activities.

Aboriginal women were recruited through community networks and were also referred from participating clinics. Information regarding the study’s aim was provided to eligible women and informed consent was obtained prior to undertaking data collection. Community stakeholders were identified through conversations with women about issues and potential priorities and were invited to participate by the study team. In Top End, women also brought Elders along to workshops. This was supplemented by stakeholder mapping exercises conducted by the study team, and additional participants were discussed with local investigators. Health professionals involved in the care of women with diabetes in pregnancy were identified at collaborating health services and were invited to participate. All participants provided written, informed consent prior to data collection. Aboriginal women and community members were provided with gift vouchers to acknowledge the participants’ contributions.

### 2.6 Data collection

The study team collected data through semi-structured workshops and interviews, each of which was attended by at least two team members. While the opportunity for discussion afforded by workshops is integral to EBCD, individual interviews were also offered to potential participants out of respect for the preference of some to share their views in a private, rather than group, setting. Interviews were also used to gather additional contextual information related to emerging priorities, or due to participant unavailability for workshops. Data collection activities were iterative, with many participants contributing to more than activity, and structured in accordance with the EBCD stages (see **Figure 2**) and resources (27). Sample workshop guides are available in the supplemental material. Planning of data collection activities were informed by Aboriginal and Torres Strait Islander investigators (KC, SG) and input from the Aboriginal and Torres Strait Islander Advisory Group, with a specific emphasis on creating a respectful space for Aboriginal women to share stories, experiences, and beliefs. This study built on our formative qualitative research exploring the experiences of NT Aboriginal women with diabetes in pregnancy (8, 9, *MacKay & Graham et al, unpublished*) and the study team used these findings to engage and further explore experiences and preferences with co-design participants. To enable participants to explore experiences, the workshops for health professionals and women/community members were facilitated separately, unless otherwise requested by women. Interviews/workshops ranged from approximately 25 minutes to two hours. None of the participants asked for an interpreter and workshops/interviews were conducted in English. The study team requested permission to audio-record interviews/workshops; in general health professionals agreed and First Nations women/community members were more likely to express reluctance. Where there was reluctance to record, the study team allocated one person to scribing and the other to facilitating.

Team members kept reflective notes which outlined additional information from workshops not evident in transcripts (i.e. non-verbal cues or relevant contextual information). Relevant information obtained through community engagement events as well as observations were noted in reflective notes, and where appropriate, queried with participants during data collection activities. These reflective notes formed an additional data source, and were particularly relevant when considering reflections on the use of EBCD. Strengths and challenges of using EBCD were also regularly considered during study meetings between authors TD, DM, KM, NB, RK and LMB, with these considerations informing the findings reported in this paper.

### 2.7 Data analysis and interpretation

Qualitative data were initially coded independently by TD, DM and either KM (Central Australian data) or NB (Top End data), using an inductive approach to identify themes. These team members then collaboratively developed mind maps to define and clarify relationships between issues and potential solutions which had emerged during initial coding. Interpretation was refined through input by study participants over time during the iterative co-design process. While qualitative data were collected from a range of informants, during data analysis and interpretation care was taken to ensure voices of Aboriginal women were elevated. To do this, the team aimed to engage with and collect data from women prior to other groups. Data from women were analysed prior to other participant groups and the priorities and concerns of women/community were used as a reference point in discussions with health/community services professionals. To arrive at the findings relating to priority issues and potential solutions, women/community priorities were privileged with data from other groups contributing to, rather than driving the discussion. Findings regarding the use of EBCD methodology arose through iterative discussions between TD, DM, KM, NB, RK and LMB.

### 2.8 Ethics

This study had prospective ethical approval from the Central Australian Human Research Ethics Committee (2021–4052) and the Human Research Ethics Committee of the Northern Territory Department of Health and Menzies School of Health Research (2021-4036). Since approval, these two committees have merged.

## 3 Findings

Participant details are shown in **Table 1**. All women and community members identified as Aboriginal; none identified as either Torres Strait Islander or both Aboriginal and Torres Strait Islander. Of the health/community service professionals, 2 of the 23 identified as Aboriginal and/or Torres Strait Islander.

### 3.1 Identification of priority issues

**Figure 3:**
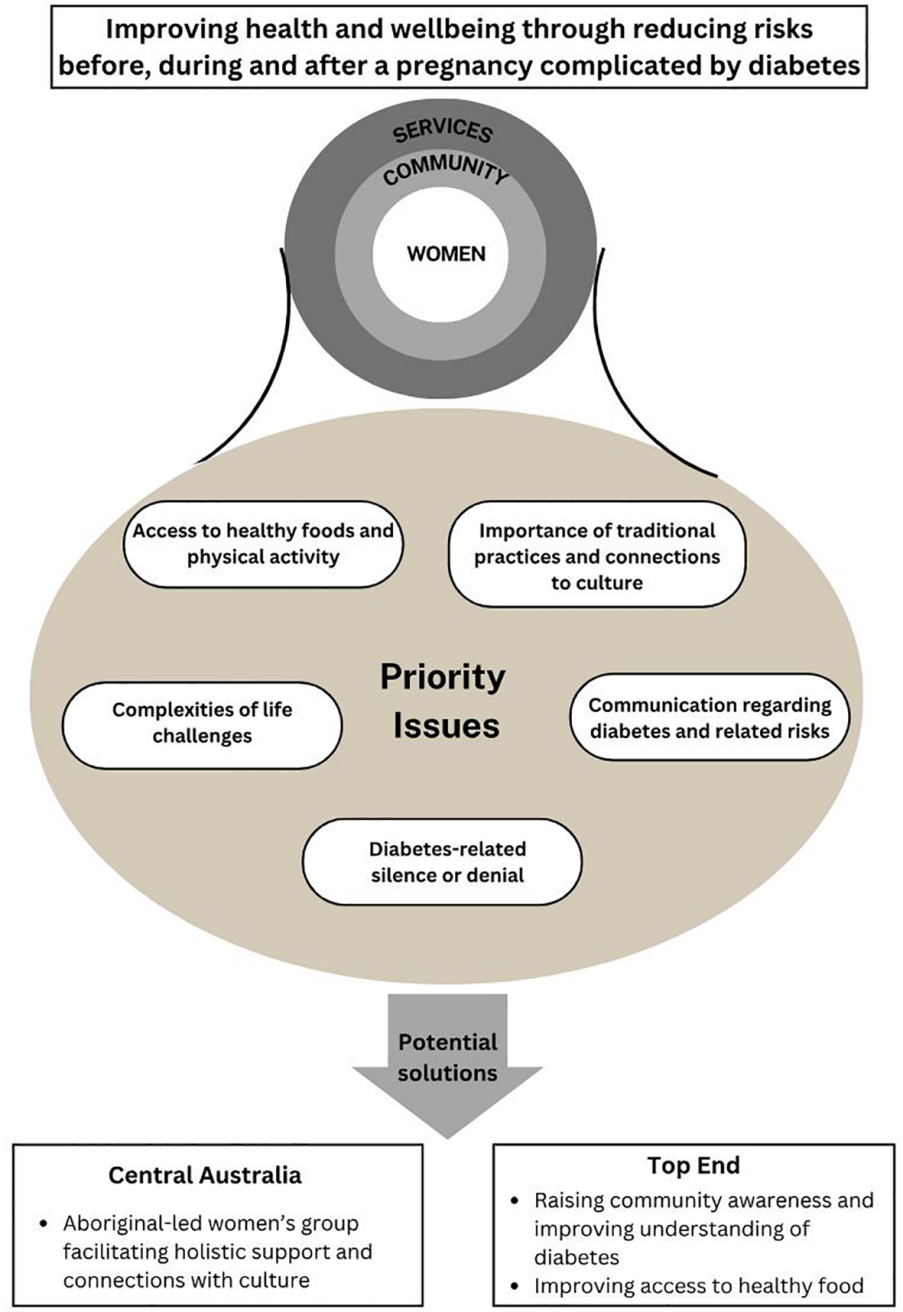
Priority issues and proposed strategies to address diabetes-related risks.

Priority issues largely aligned across both study regions and included: access to healthy foods and physical activity; the importance of traditional practices and connections to culture; communication regarding diabetes and the related risks; diabetes-related denial or silence; and other priorities women and communities face which interfere with prioritising health (**Figure 3**).

#### 3.1.1 Access to healthy foods and physical activity

While participants recognised the importance of healthy food in reducing diabetes risks, food insecurity was a common barrier: “the biggest problem here is food security and the price of food” (health professional, Top End). One participant related needing to return items after being told the cost of their groceries. Participants also expressed concerns around unhealthy dietary preferences:

“Coke is the highest selling drink in [community]. Highest selling drink. It’s crazy … they walk around with one litre bottles and just drink it. It’s really scary.” – Community member, Top End

Although participants in Top End acknowledged the role of physical activity in reducing diabetes risks, there were barriers to being active:

“Generally, a lot of the women I work with are quite active … But the weather can combat that during the wet season … at the moment it’s quite windy, so women don’t want to walk.” – Non-Indigenous Health professional, Top End

The development of a local women’s football competition was described as an opportunity for physical activity and social connection, giving women a socially accepted reason to venture from their homes.

#### 3.1.2 The importance of traditional practices and connection to culture

Engaging with traditional practices and connecting to country and culture were recognised as reducing diabetes risks:

“Some people’s diabetes stopped when they eat cultural foods and go hunting. When they do the traditional thing, not eating ‘your [Western] type of food’” – Woman, Top End

However, there were seldom opportunities for these activities due to limited transport availability. One interviewee reported the need for transport had been exacerbated by the depletion of traditional food sources nearer to the community:

“There’s too many people in this small area, taking all that bush food and so there’s nothing left for them. They need to go out further and further. So they’re looking for transport.” – Non-Indigenous Community services professional, Top End

In both study regions, women described the value of spending time with Elders to learn traditional knowledges and customs:

“I am a hunter and the young ones, they listen, they want to learn.” – Community member, Top End

#### 3.1.3 Communication regarding diabetes and related risks

The need to improve communication of health messages relating to diabetes was also expressed. Strategies to improving communication included employing more Aboriginal staff, communicating messages in local languages, and using pictorial resources:

“But anything with pictures, showing women how many sugar sachets are in things, so actually physically, if someone comes in with a can of Coke or something, and physically getting out the sugar and counting, counting, counting” – Non-Indigenous Health professional, Top End

In Central Australia, improving cross-cultural communication between health professionals and women was a priority. In the Top End, women prioritised improving community understanding of diabetes, particularly the intergenerational risks associated with diabetes in pregnancy. They highlighted that connections between diabetes in pregnancy, type 2 diabetes and diabetes complications were not well understood. Women described a need for health professionals to give “strong”, direct messages about diabetes.

#### 3.1.4 Diabetes-related silence or denial

Participants in both regions alluded to denial or a silence surrounding diabetes. In Central Australia, health professionals felt that denial was a barrier to engaging with women regarding diabetes management. One woman described not taking medications when younger as she “didn’t care” about medications then. In Top End, participants reported that diabetes in pregnancy was not talked about because women did not want to burden their families. Several participants felt that people “keep it to themselves” if they have diabetes.

“I don’t know if anybody else in my family has it. So sometimes mother one, that she go through that pregnancy diabetes or after diabetes she don’t let that daughter one know or kids know.” Woman, Top End

#### 3.1.5 Complexities of life challenges

Participants described the multiple complexities facing women and communities. These included overcrowding, housing and food insecurity, substance use, and mental health concerns. In Top End, a high rate of school non-attendance was also raised. These competing issues made it difficult for women and communities to prioritise caring for women’s health. One woman in Top End described the influence of these community issues as being “like a river flowing … You come out of the clinic and back into the river”. She was advocating the need for women to get “out of the river” to have the time and space to focus on their health. Women in Central Australia expressed needing information and support to manage life challenges. Participants reported that, while programs or services had been put in place in attempts to address some of these issues, programs were often not sustained over time:

“You hear about services and you never even knew they existed and then you go to access a service and it’s been axed. It’s gone.” – Non-Indigenous Health professional, Central Australia

Participants also highlighted the need for community programs and services to break down silos and improve communication and collaboration:

“There’s all these little pockets of programs, and I feel like we don’t talk to each other as much as we could, or the referral pathways into these different programs, everyone’s got their own thing.” – Non-Indigenous Health professional, Central Australia

### 3.2 Moving towards solutions

Emerging priority areas were fed back to participants, with opportunities to address them discussed.

In Central Australia, women expressed the desire to implement a women’s program offering holistic support before, during and after pregnancy. Through a series of workshops objectives of a program to be facilitated by an Aboriginal woman were defined and included: teaching skills and confidence, supporting the transmission of knowledge from Elders to future generations, connecting with culture through Elders, and encouraging women to support other women.

The program is also anticipated to strengthen networks between health services and other service providers, while enhancing access to services available in the study region.

The group plans to meet weekly, with sessions including a mixture of recreational, educational and social support activities. Educational topics proposed by participants include staying healthy during and after pregnancy, healthy food for mothers and families, healthy feeding for infants, managing diabetes and preventing diabetes complications. Cultural knowledges will be incorporated with each topic. It is anticipated that the schedule will include regular “Family Days”, where partners and other family members are welcome, to raise family and community awareness and strengthen family support for women.

In the Top End, the process of feeding back findings regarding priority issues and developing actions to address these issues is in progress. Participants have reiterated the need to raise community awareness and improve understandings about diabetes, specifically regarding the: high prevalence and risk factors; intergenerational impacts of diabetes in pregnancy; relationship between diabetes and the symptoms; long-term complications; and effective strategies for reducing blood sugars and improving health and wellbeing. The importance of improving the diet of community members was also emphasised. This included working with community stores to ensure availability and promotion of healthy foods and improving access to traditional foods.

During the co-design process, several implementation considerations have been identified. To ensure sustainability beyond the period of the study, the program is being designed and implemented in partnership with community organisations and structures.

“I think for a program you need longevity, so if you were going to start anything, you need something that’s going to be there a while, and longevity is based from the community.” Health professional, Top End

Additional implementation enablers and challenges are summarised in **Table 2**.

**Table 2:** Potential enablers and challenges to implementing co-designed strategies to reduce diabetes-related risks before, during and after pregnancy with Aboriginal women.

Enablers	Challenges
* Strong existing buy-in from community leaders, who are motivated to improve outcomes for their community * Embedding activities in existing organisations * Employing local community members to implement strategies * Established relationships between study team and collaborating organisations	* Limited capacity and high staff turnover of collaborating organisations * Population highly mobile/transient and at times geographically dispersed * Participants often have limited access to transport * Cultural sensitivities regarding the discussion of certain topics such as pregnancy * Diabetes-related stigma

### 3.3 Successes and challenges of EBCD methodology

The study team identified several learnings regarding the use of EBCD in this context: the utility of using EBCD while incorporating First Nations participatory principles; modifying components of EBCD to suit our context (modifications summarised in **Table 3**); prioritising stakeholder engagement; and the challenge of collaborating effectively with health services and communities despite being ‘outsiders’.

**Table 3:** Comparison of traditional experience-based co-design with hybrid approach (experience-based co-design incorporating First Nations principles).

	Experience based co-design	Hybrid approach (EBCD incorporating First Nations principles)	Rationale
Setting	Health service or health system	Engagement with Aboriginal Community Controlled Health Services as the entry point for the study team, with inclusion of community members/Elders, and community services Ongoing community engagement is essential	To ensure respect for cultural governance processes; to ensure consideration of broader social determinants of health
Stakeholders	Service users and health professionals work together; Support from organisational champions is also important	Study participants included: Aboriginal and Torres Strait Islander women (ages 16-45); Aboriginal and Torres Strait Islander community members; Community services and health professionals Ongoing reflection on dynamics and prioritising Aboriginal women’s worldviews as required Study also informed by: Study’s Investigators; Aboriginal and Torres Strait Islander leaders; and community consultation	Care was taken to balance numbers and voices of different groups and considered power dynamics Aboriginal and Torres Strait Islander leadership (academic and local) as well as input from study investigators also informed study
Sharing experiences	EBCD aims to keep consumer experience at the centre of the process and recommends: interviews, patient shadowing and observation, and filming and sharing videos of patient experiences with health professionals	De-identified stories and examples were shared between participant groups to ensure a common understanding of experiences	Privacy and cultural safety concerns meant that the study did not share stories in a video format
Dialogue and decision making	Values dialogue with mixed groups of ‘consumers’ and health professionals; uses experiences of service users to identify priorities and solutions	Dialogue happened between groups and by sharing findings between groups Information and experiences from additional stakeholders were incorporated as early findings began to emerge	availability and preferences of participants informed this structure preferences were related to concerns about cultural security and privacy
Stages	Gathering experiences; Understanding experiences; Improving the experience, and Monitoring/Maintaining experience which are implemented in an iterative manner	The study used these broad phases to structure activities	Timelines and research activities varied between sites and there were lags between phases due to contextual factors

#### 3.3.1 Use of EBCD while incorporating First Nations participatory research principles

Use of a hybrid approach of EBCD, underpinned by First Nations participatory research principles, enabled the research team to facilitate study processes with participants from three groups (Aboriginal women, community members and health professionals). EBCD’s structure guided the team and participants, while allowing for the incorporation of First Nations principles, which differ from those of the dominant Western health research paradigm.

We augmented EBCD’s approach by including community views, recognising that Aboriginal and Torres Strait Islander worldviews of health and wellbeing encompass the community. Community participants provided vital information regarding local priorities, resources and solutions, as well as community and cultural governance structures. The Central Australia study team dedicated substantial resources to networking with community services and interagency groups. This resulted in strong engagement with women, but community members were not recruited in Central Australia through this approach. The participation of community members and Elders in the Top End community was greater, as identifying and engaging Elders, cultural governance networks, and community stakeholders was straightforward in the remote context compared to the urban setting.

#### 3.3.2 Contextual modifications to EBCD methods

Storytelling is the basis of EBCD, and videos of consumers’ lived experiences are often used to spark dialogue between professionals and consumers, with an aim of transforming power imbalances between these groups. To support cultural responsivity, we modified the way participants shared stories. We did not use videos due to concerns about potential cultural inappropriateness of their use in our context. Instead, findings relating to experiences of participants from our formative research were shared verbally by facilitators as a starting point to explore the experiences of participants in the current co-design process.

Recognising the potential influence of power imbalances on workshop discussions, workshops with different participant groups (women/Elders and health professionals) were held separately rather than combined. The priorities and solutions developed by women were shared with health professionals and community stakeholders at key points. Women in Central Australia were reluctant to attend joint workshops with health professionals. Learning from that experience, the study team started discussions with Top End participants early regarding the importance of having collaborative workshops that included health professionals. Women in Top End are open to this possibility. Power dynamics between participants and health professionals are potentially different between regions, for a range of reasons. In Top End, one woman noted that she felt the clinic is open to feedback, saying that the manager’s door “… is always open for me.”

The inherent flexibility of co-design approaches allowed the study team to respond to women’s preferences. For example, at Top End, women requested a shift from a focus on diabetes in pregnancy to holistic women’s health, to better align with community and cultural norms. The study team worked with women to rename the study, reframe key messages, and to implement strategies to ensure that women with diabetes in pregnancy and those at risk of diabetes were still included. Similarly, while there has been broad alignment of priority issues between both study sites, the flexibility of co-design has ensured that strategies for implementation are being shaped by the context of each site, particularly based on the perspectives of the Aboriginal women participating.

#### 3.3.3 Prioritising stakeholder engagement

Understanding the necessity of building trust with participants, the team prioritised engagement with women and community members prior to data collection. In Central Australia, the team attended and hosted health promotion events to build rapport with potential participants. Similarly, the Top End team visited the site three times, for several days at a time, prior to any data collection. This time was used to promote the project, and to identify and opportunistically engage with stakeholders (both in community and when stakeholders were in Darwin). Effective collaboration required consideration in scheduling research activities, participant availability, clinic/community capacity and other events (i.e. Sorry Business). In between data collection activities, Central Australian team members informally visited participants and supported participants to access recreational and educational community services. The team working with the Top End site communicated frequently via phone and email between study visits. This highlights that, for EBCD to be used effectively, substantial resources need to be committed to stakeholder engagement activities and these duties need to be undertaken with sensitivity and flexibility.

Despite these efforts, the team was challenged by the changing membership of the participant groups. Over the period (20 months in Central Australia and nine months in Top End), some women moved away or were unable to maintain participation in the post-partum period. This lack of continuity presented a challenge to the study team as it often meant re-capping previous conversations and integrating additional views, which required an adjustment of the study team’s expectations regarding timelines. Changing membership, however, enabled the inclusion of additional views. The team found that building relationships with health professionals in each site also required time. Some health professionals, already managing high workloads, were initially skeptical about the project, voicing concerns about potential limited impact in the face of the overwhelming inequities faced by Aboriginal and Torres Strait Islander women. It was observed that once engaged, however, health professionals were energised by their involvement.

#### 3.3.4 Effective collaboration with services and communities when an “outsider”

The research team being external to the health services and communities presented challenges. While partners were invited to participate based on previous shared collaborations for over a decade, the co-design methodology of this study differed significantly from prior collaborative work and required deeper engagement. Long-term ownership was considered from the outset, with internal guidelines established to maximise the decision-making power of study participants during the co-design process, whilst recognising the need for clinic/community support, and also negotiating existing constraints related to funding deliverables and timelines. The study team navigated local governance processes as the study moved towards designing solutions. In Central Australia, it was important to liaise with researchers working on other projects in similar fields, such as maternal health, to ensure that participants were not overburdened. The study team progressed research activities while understanding and respecting priorities faced by health services/communities.

## 4 Discussion

We have described the use of a hybrid co-design approach in the remote Northern Australian context to identify strategies to reduce risks before, during and after a pregnancy complicated by diabetes. We have reported the early findings from this co-design process, which will inform the strategies to be implemented. Key findings include the relationship between traditional cultural practices and wellbeing, the need for improved communication relating to diabetes and related risks, and the impact of the complexities of women’s lives on their ability to prioritise caring for their own health. We have also described learnings from our use of a hybrid co-design approach; these learnings have implications for similar participatory practices in the remote Australian context, particularly in the primary care/community setting, as well as being potentially relevant to projects working with First Nations populations or groups experiencing disadvantage more broadly.

The contribution of traditional hunting and feeding practices, and, conversely, that of Western eating practices, to the metabolic health of Aboriginal people has long been understood (28). In our study, participants also clearly articulated this relationship, while expressing the desire to have increased opportunities to engage in traditional practices. These findings align with and build upon findings from our previous work, which informed this co-design study, in which Aboriginal women described improved physical and mental health when connected to Country (9). Previous studies in Australia and Canada have identified benefits in biomedical, psychosocial and social health through engagement in Aboriginal Land Management activities (29).

The need for improved communication about diabetes has also been reported in previous studies (8, 30, 31). In our previous phenomenological study, which informed the current co-design study, Aboriginal women consistently emphasised the importance of cultural appropriateness when sharing information about diabetes in pregnancy (8). Our co-design study has extended this finding, particularly through the emphasis placed on improving community awareness of the intergenerational risk of diabetes in pregnancy. Raising community awareness is an important element of addressing diabetes-related silence or denial, which has also been reported among young Aboriginal people with T2D (30). Improving communication to ensure a deep understanding of diabetes-related messages among Aboriginal communities is crucial; this understanding is a necessary component of empowering Aboriginal people to take action to reduce the risks associated with diabetes (32).

In our study, women’s health was frequently de-prioritised due to competing issues. In both urban and remote settings, food and housing insecurity are distressingly common for Aboriginal people (33). In the context of poverty, people preferentially purchase energy dense, nutrient poor food, increasing the risk of diabetes (34). In addition to dealing with food and housing insecurity, Aboriginal women who have had diabetes in pregnancy often have caring responsibilities for children and unwell family members, and prioritise the needs of family above their own health (9, 10). There is a pressing need for systemic action to address the social determinants of health, while additionally supporting Aboriginal women to navigate the many priorities they face. There is also a need for holistic services and programs to support women which are accessible, culturally safe and sustained beyond the current pattern of often short-term funding cycles. Aboriginal community-controlled services are well placed to provide these supports but require additional resourcing and supports (35).

The above key findings were identified through a hybrid co-design process. There is considerable ambiguity surrounding the term ‘co-design’ (36), wide variations across projects (37), and often limited transparency regarding structures and processes (15, 38). This paper addresses these gaps while adding to the existing literature on EBCD, which includes examples in the remote Northern Australian context (39) and with multiple stakeholders and sites (40). EBCD was successfully used by Raynor et al. to define intervention components which were later evaluated (40). The EBCD process in our study had an additional strength of exploring implementation considerations, providing the opportunity for the study team to plan to leverage identified enablers while considering contingencies for identified barriers.

We modified EBCD to support genuine engagement of Aboriginal women and community members. Collaborative approaches are essential to avoid replicating colonial histories, which have centred and normalised Western biomedical approaches as the authority, while devaluing other worldviews and knowledges, reinforcing inequitable power dynamics (41). Dialogue with communities is essential for ethical and effective health promotion with Aboriginal and Torres Strait Islander people (42) and supports program sustainability and positive health outcomes (43). These partnerships require the voices and contributions of all parties to be respected (44). The importance of building trust, genuine partnerships, and respectful collaboration with local stakeholders is also echoed in the international public health co-design literature (45). This respect is particularly important as during such partnerships tension can arise between responsiveness to community priorities and ensuring the pre-specified project aim is met. Ideally, community-based research projects would be informed by co-design processes from inception, ensuring community priorities and study aims are aligned; however, even in an ideal scenario, adaptation to co-design during the process may be required according to the needs of participants. In our study, broadening the focus of the program at the Top End site from diabetes in pregnancy to a more holistic view of women’s health was an opportunity to respond to community preferences and ensure acceptability in a way that still aligned with the study aim.

Genuine engagement, despite being identified as a key enabler for interventions aiming to improve the health of Aboriginal and Torres Strait Islander peoples, is often curtailed due to restrictions on time and resources (46). Modifications to EBCD in this study align with best practice approaches for health co-design projects with Aboriginal and Torres Strait Islander people and communities (47). Our study meets health research quality standards articulated from an Aboriginal and Torres Strait Islander perspective, including elevating the voices of Aboriginal community members and embedding Aboriginal leadership, which is the basis of this study’s governance structure (48). The inclusion of local Aboriginal women in the research team, who contributed to participant recruitment and workshop facilitation, was a key strength of our study, enabling meaningful engagement with participants and enhancing accurate interpretation of data.

Diabetes and other chronic conditions urgently require preventative and collaborative approaches. The under resourcing of remote primary care in the remote NT context (49) and workforce shortages (50) require not only additional resources but also innovative approaches that engage communities. Our early findings point to the possibility of exploring co-design partnerships between researchers and primary care health services, potentially enhancing community engagement with the robust and skilled continuous quality improvement primary care workforce in the NT (51). However, we have also highlighted the resource-intensive nature of conducting co-design processes. Given the current resource limitations on primary care health services in the NT, further investment would be required to yield the potential benefits of co-design partnerships.

Participatory approaches are theorised to enhance outcomes for consumers, build capacity through shared learning (52), shift power dynamics (15) and address inequities (18). It is worth noting that shifting power dynamics requires not only efforts to engage but also truly listen to First Nations consumers and communities and act together; that the “right conditions” need to be in place for different groups to learn, share and create together (52). Supporting genuine participation, however, is only a first step, and women in this study likely experienced a range of barriers, potentially including gender dynamics, the shame/stigma of diabetes, which has been previously reported in the NT among young Aboriginal people with T2D (30), not to mention the impacts of structural racism experienced by First Nations people (5). The study team took steps, as described earlier, to address these barriers where possible and create a safe environment for participants, although acknowledge one limitation of this study being barriers which are either unknown or too pervasive for the team to address. Whether or not the conditions have been created in this study to enable the potential of participatory approaches remains to be examined in the study’s next phase.

Our study was affected by several other limitations. These included the inability to audio record and transcribe several workshops due to participant preferences. However, respecting these preferences was viewed as essential, and the study team took as detailed notes as possible, with key findings confirmed with participants at subsequent workshops. Another limitation was the loss of momentum that occurred due to long periods of time passing between some data collection and engagement activities, often due to health services or community members having limited capacity to support research activities. This underscores that health services and communities need to be adequately supported to engage in research for such collaborations to be successful. We anticipate that strategies implemented at each study site as a result of this co-design process will differ, meaning that direct comparisons between site outcomes will not be possible. However, direct comparison of results between sites is not the objective of our overall study; rather, we will aim to evaluate the outcomes and implementation of strategies within each site’s own specific context. The implementation of contextually relevant strategies at each site should be viewed as a strength, and can be expected to enhance community engagement and sustainability; this will be assessed when evaluating implementation.

To conclude, use of EBCD methodology with the incorporation of First Nations participatory principles has enabled the identification of shared priorities and strategies to reducing diabetes-related risks before, during and after pregnancy. While priority issues were consistent across study regions, the flexibility of EBCD ensured the strategies developed are informed by the local context. Challenges experienced with the use of EBCD included maintaining meaningful engagement and collaborating effectively with services and communities when an “outsider”. Lessons outlined in this paper may support others when considering mechanisms to strengthen consumer and community engagement in primary care.

## Data availability statement

The datasets presented in this article are not readily available because primary qualitative data may include contextual details which could identify participants and therefore will not be available. The study team would consider reasonable requests to view documents that contain synthesized findings. Requests to access the datasets should be directed to diana.mackay@menzies.edu.au.

## Ethics statement

This study involved humans and was approved by the Central Australian Human Research Ethics Committee and the Human Research Ethics Committee of the Northern Territory Department of Health and Menzies School of Health Research. The study was conducted in accordance with the local legislation and institutional requirements. The participants provided their written informed consent to participate in this study.

## Author contributions

TD: Formal analysis, Investigation, Methodology, Project administration, Writing – original draft, Supervision. DM: Conceptualization, Formal analysis, Methodology, Supervision, Writing – original draft, Funding acquisition, Investigation. KC: Conceptualization, Formal analysis, Funding acquisition, Methodology, Writing – review & editing, Supervision. JB: Conceptualization, Formal analysis, Methodology, Supervision, Writing – review & editing, Funding acquisition, Resources. HD’A: Formal analysis, Supervision, Writing – review & editing. DH: Conceptualization, Formal analysis, Supervision, Writing – review & editing. KM: Formal analysis, Investigation, Project administration, Writing – review & editing. JP: Formal analysis, Investigation, Resources, Writing – review & editing. NB: Formal analysis, Investigation, Project administration, Visualization, Writing – review & editing. HM: Conceptualization, Formal analysis, Funding acquisition, Methodology, Writing – review & editing. SG: Conceptualization, Formal analysis, Methodology, Supervision, Writing – review & editing, Investigation. SC: Conceptualization, Formal analysis, Funding acquisition, Supervision, Writing – review & editing. CC: Conceptualization, Funding acquisition, Writing – review & editing. LM: Conceptualization, Funding acquisition, Writing – review & editing. RK: Conceptualization, Formal analysis, Funding acquisition, Investigation, Methodology, Supervision, Writing – review & editing. LMB: Conceptualization, Formal analysis, Funding acquisition, Investigation, Methodology, Resources, Supervision, Writing – review & editing.
