# Supplementary material for: Supporting healthy lifestyles for First Nations women and communities through co-design: lessons and early findings from remote Northern Australia
Source: Front Clin Diabetes Healthc. 2024 May 28;5:1356060. doi: 10.3389/fcdhc.2024.1356060 (PMC11165116; doi:10.3389/fcdhc.2024.1356060)
Supplement: Supplementary file 1 [file Supplementary.zip › Supplementary/Supp1 Example Workshop Guide.docx]

**Supplemental Material 1: Sample workshop guides**

**ENGAGE workshops: build relationships with participants and commence study**

Activities with participants (women and community members) may include:

- Informal and social activities to build trust and rapport (presence at regular activities and events in the community) between participants and study team
- Accessible ways to provide information about study and diabetes and to support informed consent
- Discussion of co-design approaches and differentiate from other research approaches
- Stakeholder mapping

*Guiding questions for women and community:*

- What do you know about diabetes?
- What do you know about diabetes in pregnancy?
- Who has given you information about diabetes?
- What keeps you well and strong?
- How does diabetes impact your community?
- How do people talk about diabetes in your community?
- What is your community doing to stay healthy?
- What has been your experience of research in your community?
  - What was good?
  - What was not so good?
- What do you think is important for a good co-design process?

*Guiding questions for health and community services professionals:*

- What motivates you to work with women with diabetes?
- What frustrates you about working with women with diabetes?
- What does your service do well?
- What specific things enable women to access your service?
- What areas could your service improve on?
- What are the barriers to women accessing your service?
- How will your service look in the future?
- What community-based programs or changes would help support women with diabetes in pregnancy?
- What community-based programs or changes would help prevent women developing diabetes before, during and after pregnancy?
- Do you have any ideas about health promotional programs that may work here?
- In terms of supporting women to be healthy before, during and after pregnancy – we have heard people talk about food security being an issue – is that your observation too?
- Can you think of anything that could support women & the community to strengthen food security?
- Any observations around physical activity in the community for women? What’s working well and anything that could be improved?

**GATHER workshops: gain a deeper understanding of experiences**

Activities with participants to better understand individual and community experiences. May include:

- Reflecting on discussion from ENGAGE workshop and further discussing specific points
- Discussing relevant research findings (i.e. ‘stories’ from formative work) to see how this compares to experiences
- Through conversations, commence drawing out themes and checking with participants analysis
- Sharing information between participant groups

*Guiding questions for women/community:*

- What are the issues in your community? Do you think that diabetes is a priority?
- Can you tell us about the leaders in the community? Do you think they would be interested in this project?
- We have that (share anecdote/experience): is this true for you? In your community?
- Did any of these stories make you think of yourself or someone else you know?
- What were the similarities to these stories and your experience/the experiences of people you know?
- What were the differences?
- We’ve heard that (X) is working well, can you tell us more about this?
- From our discussion last time, it sounds like (X) may be a challenge – can you tell us more about this?
- What are the resources required to address diabetes in this community?

*Guiding questions for health and community services professionals:*

- We have mapped out what we’ve heard from health/community services professionals and we’ve put them in this mind map
- Based on your experiences: have we missed anything? Are there other concerns or issues we should add?
- Based on the emerging priorities, these are some of the solutions that were raised…what have we missed? What should we add? Are any of these already happening? What is the most important to you?
- Women and community members have told us that their priorities are (X, Y, Z) – what are your thoughts?

**UNDERSTAND stage: discuss findings, identify opportunities and priorities for change**

Discussions and activities at this stage are focussed on confirming analysis, discussing priorities for change, potential solutions (including enablers and barriers)

Activities were enhanced by visual prompts and activities to support active engagement and dialogue (mind maps, ways for participants to vote).

Additional information was requested from health/community service professionals regarding specific priorities and solutions

*Guiding questions for women and community members:*

- We would like to discuss the information you have provided in the previous workshop to ensure what we have recorded is appropriate.
- It sounds like (A, B, C, D, E) are the priority issues here, is this accurate?
- We have started mapping out the issues and solutions, what have we forgotten? Is there anything we should change?
- What’s most important – what should be prioritised?

*Guiding questions for health/community services professionals:*

- Discuss emerging priorities and potential solutions identified by women/community and intersection with health/community services professionals
- Discussion of emerging priorities: is there anything we should add? Anything that’s been left out?
- Discussion of solutions: existing service plans and potential plans
