# Supplementary material for: Supporting healthy lifestyles for First Nations women and communities through co-design: lessons and early findings from remote Northern Australia
Source: Front Clin Diabetes Healthc. 2024 May 28;5:1356060. doi: 10.3389/fcdhc.2024.1356060 (PMC11165116; doi:10.3389/fcdhc.2024.1356060)
Supplement: Supplementary file 1 [file Supplementary.zip › Supplementary/Supp2 Table - EBCD activities.docx]

**Supplementary material 2: Timeline and details of EBCD activities**

| **CENTRAL AUSTRALIA activities** | | | |
| --- | --- | --- | --- |
| **Date** | **EBCD Stage and objective** | **Format** | **Participants** |
| April 2022 | ENGAGE health professionals: provide information to clinic about project and identify interested participants | Presentation and discussion to health professionals | - |
| April 2022-August 2022 | ENGAGE: Weekly attendance at women’s activities at the clinic; to engage with and build relationships with women | Engagement with potential participants | - |
| July 2022 | GATHER workshop: to understand health professionals experiences and views of diabetes in pregnancy in Central Australia | Workshop | 6 health professionals |
| August 2022 | ENGAGE: meetings with key allied health professionals | Introducing the study to key allied health professionals |  |
| August 2022 | UNDERSTAND workshop with health professionals reflect on information from previous workshop (GATHER); discuss themes, identify opportunities for change and priorities. Share findings of formative work (Voices of Aboriginal and Torres Strait Islander women with diabetes in pregnancy) | Workshop | 8 health professionals |
| November 2022 | ENGAGE: workshop with Aboriginal and Torres Strait Islander women; to understand experiences of diabetes (personal experiences and family experiences) | Workshop | 3 women |
| December 2022 | GATHER: workshop with Aboriginal and Torres Strait Islander women to reflect on and understand experiences and to begin to identify priorities. | Workshop | 3 women |
| May 2023 | ENGAGE: Re-engagement with health professionals, necessary due to time lags and staff turn over | Presentation and discussion |  |
| June 2023 | ENGAGE: engagement event hosted by study team at the clinic site. To re-connect with participants and to recruit additional participants after delays | Engagement activity to update participants, to introduce project to potential participants and to update health professionals. Was attended by approximately 15 women and 11 health professionals |  |
| June 2023 | UNDERSTAND workshop | Series of workshops with women to discuss:   - Identifying priorities - Further shaping of women’s program including priority topics - Discussing barriers and enablers | 4 women, 2 community services professionals |
| June 2023 | UNDERSTAND workshop |  | 6 women, 1 community services professional |
| June 2023 | UNDERSTAND workshop |  | 5 women, 1 community services professional |
| June 2023 | UNDERSTAND workshop Discussion of emerging program concept; discussion regarding service alignment and other implementation considerations | Workshop | 9 health professionals |

| **TOP END activities** | | | |
| --- | --- | --- | --- |
| **Date** | **EBCD Stage and objective** | **Format** | **Participants** |
| February 2023 | ENGAGE: Principal Investigator travelled to study site to meet key stakeholders | Discussions and meetings – not data collection |  |
| March 2023 | ENGAGE: study team travelled to site to meet with key stakeholders | Discussions and meetings – not data collection |  |
| Early May 2023 | ENGAGE: meetings with local Aboriginal Leaders; information session and consultation session with female community members | Discussions and meetings – not data collection |  |
| Late May 2023 | GATHER: to understand personal and community views of diabetes and diabetes in pregnancy | Interview | Community member |
|  | GATHER: to understand personal and community views of diabetes and diabetes in pregnancy | Interview | Community member (Elder) |
|  | GATHER: to understand health professionals experiences and views of diabetes in pregnancy in community | Workshop | 3 health professionals |
|  | GATHER: to understand experiences and views of diabetes in pregnancy in community | Interview | 1 health professional |
| June 2023 | GATHER: to understand experiences and view of diabetes and diabetes in pregnancy in the community | Workshop | 3 women, 2 Elders/community members |
|  | GATHER: to understand experiences and view of diabetes and diabetes in pregnancy in the community | interview | 1 woman |
| October 2023 | UNDERSTAND: check information provided in previous workshop, discuss emerging priorities and opportunities  Discussion of emerging priorities and next steps | Workshop | 3 women, 4 Elders/community members |
|  | UNDERSTAND: check information provided in previous interview, discuss emerging priorities and opportunities | Interview | 1 health professional |
|  | UNDERSTAND: check information provided in previous interview, discuss emerging priorities and opportunities | Interview | 1 Community member |
|  | UNDERSTAND: check information provided in previous workshop, discuss emerging priorities and opportunities | Interview | 1 health professional |
|  | GATHER/UNDERSTAND: discussion of project and emerging themes. Discussion of priorities relevant to this stakeholder and opportunities moving forward | Interview | 1 Community services professional |
